# Older adults at greater risk for Alzheimer’s disease show stronger associations between sleep apnea severity and verbal memory

**DOI:** 10.21203/rs.3.rs-3683218/v1

**Published:** 2023-12-02

**Authors:** Kitty Lui, Abhishek Dave, Kate Sprecher, Miranda Chappel-Farley, Brady Riedner, Margo Heston, Chase Taylor, Cynthia Carlsson, Ozioma Okonkwo, Sanjay Asthana, Sterling Johnson, Barbara Bendlin, Bryce Mander, Ruth Benca

**Affiliations:** San Diego State University/University of California San Diego, Joint Doctoral Program in Clinical Psychology, San Diego, CA, USA; Department of Cognitive Sciences, University of California, Irvine; Department of Population Health Sciences, University of Wisconsin-Madison; Department of Neurobiology and Behavior, University of California, Irvine; Department of Psychiatry, University of Wisconsin-Madison; Department of Medicine, University of Wisconsin-Madison; Department of Neuroscience, University of Kentucky; Wisconsin Alzheimer’s Disease Research Center, University of Wisconsin-Madison; Wisconsin Alzheimer’s Disease Research Center, University of Wisconsin-Madison; Wisconsin Alzheimer’s Disease Research Center, University of Wisconsin-Madison; Wisconsin Alzheimer’s Disease Research Center, University of Wisconsin-Madison; Department of Medicine, University of Wisconsin-Madison; Department of Psychiatry and Human Behavior, University of California, Irvine; Department of Psychiatry and Behavioral Medicine, Wake Forest University

## Abstract

**Background:**

Obstructive sleep apnea (OSA) increases risk for cognitive decline and Alzheimer’s disease (AD). While the underlying mechanisms remain unclear, hypoxemia during OSA has been implicated in cognitive impairment. OSA during rapid eye movement (REM) sleep is usually more severe than in non-rapid eye movement (NREM) sleep, but the relative effect of oxyhemoglobin desaturation during REM versus NREM sleep on memory is not completely characterized. Here, we examined the impact of OSA, as well as the moderating effects of AD risk factors, on verbal memory in a sample of middle-aged and older adults with heightened AD risk.

**Methods:**

Eighty-one adults (mean age:61.7±6.0 years, 62% females, 32% apolipoprotein E ε4 allele (*APOE4*) carriers, and 70% with parental history of AD) underwent clinical polysomnography including assessment of OSA. OSA features were derived in total, NREM, and REM sleep. REM-NREM ratios of OSA features were also calculated. Verbal memory was assessed with the Rey Auditory Verbal Learning Test (RAVLT). Multiple regression models evaluated the relationships between OSA features and RAVLT scores while adjusting for sex, age, time between assessments, education years, body mass index (BMI), and *APOE4* status or parental history of AD. The significant main effects of OSA features on RAVLT performance and the moderating effects of AD risk factors (i.e., sex, age, *APOE4* status, and parental history of AD) were examined.

**Results:**

Apnea-hypopnea index (AHI), respiratory disturbance index (RDI), and oxyhemoglobin desaturation index (ODI) during REM sleep were negatively associated with RAVLT total learning and long-delay recall. Further, greater REM-NREM ratios of AHI, RDI, and ODI (i.e., more events in REM than NREM) were related to worse total learning and recall. We found specifically that the negative association between REM ODI and total learning was driven by adults 60+ years old. In addition, the negative relationships between REM-NREM ODI ratio and total learning and REM-NREM RDI ratio and long-delay recall were driven by *APOE4* carriers.

**Conclusion:**

Greater OSA severity, particularly during REM sleep, negatively affects verbal memory, especially for people with greater AD risk. These findings underscore the potential importance of proactive screening and treatment of REM OSA even if overall AHI appears low.

## Background

Obstructive sleep apnea (OSA) is characterized by recurrent pharyngeal airway collapses that cause complete (apneas) or partial (hypopneas) cessations of airflow that lead to sleep fragmentation and intermittent hypoxemia[1]. Meta-analyses have shown that sleep-disordered breathing (SDB), such as OSA, greatly increases incidence of Alzheimer’s disease (AD), and people with AD were more likely to have OSA[2,3]. OSA exacerbates AD pathophysiology, medial temporal lobe (MTL) degeneration, and memory impairment, which could likely be driven by OSA-related hypoxemia[4–8], although some have reported that the cognitive consequences of OSA are diminished in older age[9]. The exact OSA features driving these relationships have remained unclear. Moreover, even though the influence of age, sex, and apolipoprotein E ε4 (*APOE4*) genotype are known moderators of OSA expression, their influence on the relationships between OSA and memory function is not well studied[10–13].

OSA and AD are both more prevalent in the aging population and are linked to cognitive impairment, but this relationship may be modified by known AD and OSA risk factors[13]. 40–80% of people with AD carry at least one *APOE4* allele and older adults with *APOE4* may have increased risk for SDB, although this has not been consistently reported[12,14]. Sex differences are also observed in AD and OSA risk. Women are nearly twice as likey to be diagnosed with AD with a more severe presentation with faster memory decline and more pathological tau accumulation [15–18]. While men are at increased risk for OSA in younger and middle-age, OSA prevalence substantially rises in post-menopausal women[11,19]. OSA is also expressed differentially by sex, with apneas and hypopneas more likely to occur in rapid eye movement (REM) sleep in women[20,21].

Some evidence indicated that OSA severity, specifically during REM sleep, may impart greater cognitive and neurodegenerative consequences, potentially due to the greater neurometabolic demand in AD-sensitive regions in REM sleep relative to non-rapid eye movement (NREM) sleep[22–24]. During REM sleep, there is an increased susceptibility of upper airway collapse due to inhibition of the genioglossus muscle (the major upper airway dilator muscle that helps stabilize breathing)[25]. There are also lower hypoxic and hypercapnic respiratory drives during REM which result in longer durations of apneas and hypopneas, and more instances of oxyhemoglobin desaturation[26]. However, little is known about the effects of REM OSA on memory and AD risk.

Here, in the current study, we sought to examine the relationships between sleep stage-specific OSA expression and memory function, and whether AD risk factors moderate these relationships. We combined clinical polysomnography (PSG) with verbal memory measured by the Rey Auditory Verbal Learning Test (RAVLT) in a cohort of middle- and older-aged adults enriched for parental history and genetic risk for AD. We aimed to extend the current literature by testing the following hypotheses: 1) greater OSA severity, particularly during REM sleep, is associated with impaired verbal learning and recall and 2) this relationship is accentuated among women, older adults, and/or individuals with increased genetic and parental risk for AD.

## Methods

### Clinical methods

Eighty-one cognitively unimpaired middle- and older-aged adults participated in a cross-sectional study who enrolled in the Wisconsin Alzheimer’s Disease Research Center (ADRC) Clinical Core, a prospective cohort study enriched for probable parental history of AD relative to the general population[27]. *APOE4* was genotyped by DNA extraction from whole blood samples using competitive allele-specific PCR based KASP genotyping for rs429358, as previously reported[28]. This study excluded for individuals with a past history or current neurological, psychiatric, medical conditions, or treatments that impacted their cognition, or hindered their ability to complete any aspects of the study protocol, taking medications known to influence sleep or sleep electroencephalography (EEG), including antipsychotic medications, non-selective serotonin reuptake inhibitors (SSRIs) antidepressants, neuroleptics, chronic anxiolytics, sedative hypnotics, and stimulants, and was currently undergoing treatment for SDB (e.g. continuous positive airway pressure). Participants underwent cognitive assessments of declarative and semantic memory, attention, executive function, language, and visuospatial processing using the National Alzheimer’s Coordinating Center Uniform Data Set (UDS) neuropsychological battery version 3 and additional assessments[29]. Clinical diagnosis of cognitively unimpaired status was determined using the 2011 National Institute on Aging-Alzheimer’s Association (NIA-AA) workgroup diagnostic criteria and confirmed by multidisciplinary consensus conference **(Table S1)**[30,31]. Polysomnography (PSG) with high-density EEG (hdEEG) was performed within 1 year of cognitive assessment.

### Polysomnography

To assess sleep and OSA severity, participants underwent clinical PSG with 256-channel hdEEG. A thorough description of PSG with hdEEG recording and sleep scoring has been previously described[32]. From PSG, sleep architecture measures of total sleep time (TST), time in bed (TIB), sleep onset latency, wake after sleep onset (WASO), and percent of TST spent in N1, N2, N3, and REM were derived. Additionally, clinical measures reflecting sleep disorder characteristics were calculated, including apnea-hypopnea index (AHI; number of apneas and hypopneas per hour), respiratory disturbance index (RDI; number of apneas, hypopneas, and respiratory-related arousals per hour), oxyhemoglobin desaturation index (ODI; number of oxyhemoglobin desaturations ≥4% per hour), nadir blood oxyhemoglobin saturation level, mean blood oxyhemoglobin saturation, duration of time spent at <90% blood oxyhemoglobin saturation, and periodic leg movements during sleep index (PLMSI; number of PLMs per hour). AHI, RDI, ODI, and duration of time spent at <90% oxyhemoglobin saturation in REM and NREM sleep were also derived. To measure whether an individual has more REM or NREM OSA features throughout the night, ratios of AHI, RDI, and ODI between REM and NREM sleep were also calculated[33,34].

### Rey Auditory Verbal Learning Test (RAVLT)

The RAVLT is a standard neuropsychological assessment for verbal memory that it MTL-dependent, a sensitive marker of memory impairment in preclinical AD, and commonly used in AD research and clinical practice for diagnosis [35–38]. OSA severity has been linked to verbal memory performance, and learning and recall of verbal information is highly sleep-dependent. [8,39,40].

The test includes one learning phase, two recall phases, and one recognition phase. During the learning phase, a list of 15 words is read to the participant five times, and the participant repeats the word they remember after each trial. A total learning score is derived by summing the number of remembered words in trials 1 through 5. An interference list of 15 words is then read aloud once, and the short-delay recall ability is assessed after the interference list. Long-delay recall is then assessed after 20 minutes.

### Statistical analyses

The OSA characteristics that were analyzed were AHI, RDI, and ODI, and sleep duration spent with <90% blood oxyhemoglobin saturation for total sleep, REM sleep, and NREM sleep. Total sleep nadir blood oxyhemoglobin saturation, total sleep mean blood oxyhemoglobin saturation, and WASO were also analyzed. RAVLT measures included total learning, short-delay recall, and long-delay recall. Normality of variables were analyzed with a Shapiro-Wilk test. All AHI, RDI, and ODI measures were log-transformed with a constant added to meet normality assumptions.

*APOE4* status was not obtained for one participant and was not included in any statistical analysis with *APOE4* status. Independent sample t-tests were conducted to analyze group differences by sex, *APOE4* status, and parental history of AD. Student’s t-tests were used if assumptions of normality and variance were met. Mann Whitney U-tests were used if assumptions of normality were violated. Kendall rank correlation was conducted on the associations between age and OSA characteristics. Paired samples t-tests were used to analyze REM versus NREM AHI, RDI, ODI, and duration spent with <90% blood oxyhemoglobin saturation. Student’s t-test was used if assumptions of normality and Wilcoxon signed-rank test was used if assumptions of normality were violated.

Multiple linear regression models were used to analyze the relationships between OSA characteristics (predictors) and RAVLT (outcomes), controlling for sex, age, time between PSG and RAVLT, *APOE4* status, body mass index (BMI), and years of education. Across all 15 models, the Benjamin-Hochberg method for False Discovery Rate (FDR) correction was used to correct multiple comparisons[41]. Regressions were repeated, substituting *APOE4* status for parental history of AD.

To understand the relative impact of OSA during REM sleep against NREM sleep on verbal memory performance, post hoc analyses included using the Steiger’s Z test to directly compare the correlation strengths of the associations between REM and NREM sleep apnea features and RAVLT scores [42]. Also, REM-NREM AHI, RDI, and ODI ratios were calculated and the ratios were log-transformed with a constant added to meet normality assumptions. Multiple linear regression models were then used to analyze the associations between the OSA feature by sleep stage ratios and RAVLT scores while controlling for the same covariates. Regressions were repeated substituting *APOE4* status for parental history of AD.

Moderation analyses were also conducted by probing the interaction between sleep apnea characteristics with sex, age, *APOE4* status, and parental history of AD (*APOE4* status and parental history of AD in separate regression models). To further probe significant interactions with either *APOE4* status or parental history of AD, we grouped participants into three groups consisting of 1) people with no *APOE4* or parental history of AD, 2) people with either *APOE4* or parental history of AD, and 3) people with both *APOE4* and parental history of AD. Analysis of covariance (ANCOVA) was used to analyze interactions between AD risk group and OSA features as it predicted verbal memory while controlling for the same covariates mentioned above. Slopes of the relationship between OSA features and verbal memory between the 3 groups were compared and Tukey’s method was used to correct for multiple comparision[43]. For significant interactions with age, Johnson-Neyman intervals and simple slope analyses were used to determine how much of the sample was driving the significant moderating effect[44–46]. All statistical analysis was conducted on JASP (Version 0.17.3) and RStudio (Version 2021.09.2)

## Results

### Sample characteristics

Participant demographics and RAVLT scores of the 81 participants are shown in Table 1. Sleep architecture characteristics are shown in Table 2. In this sample, the average age was 61.7±6.0 years (age range: 44–88 years), 62.0% participants were female, 31.7% of them were *APOE4* carriers, 69.5% had parental history of AD, and 26.3% were both *APOE4* carriers and had parental history of AD. The average time between PSG and RAVLT assessments was 0.31±0.50 years. Nearly half of the cohort (44.44%) had OSA (AHI≥5/h) and 16.05% had moderate or severe OSA (AHI≥15/h). OSA severity was significantly higher in REM sleep than in NREM sleep (AHI: (t(80)=7.91, p<0.001), RDI: (t(81)=5.11, p<0.001), ODI: (t(81)=9.64, p<0.001). Remarkably, the duration spent with blood oxyhemoglobin levels <90% did not differ significantly between REM and NREM sleep stages (1.83±4.57 versus 2.55±7.21, z=0.06, p=0.96), despite the fact that participants spent significantly more of the sleep period in NREM than in REM sleep stages (NREM:279.49±57.71 mins versus REM:59.80±28.69 mins, t(80)=36.17, p<0.001). This resulted in the proportion of time spent with blood oxyhemoglobin levels <90% being significantly higher during REM than in NREM sleep stages (REM: 0.04±0.11 versus NREM:0.01±0.03, z=3.68, p<0.001).

### Sex, age, *APOE4 status*, parental history of AD effects on OSA

Overall, male participants had more severe OSA than females (**see Table S2**). However, females did have a significantly higher REM-NREM ODI ratio (t(79)=2.45, p=0.02). There were no significant associations observed between age and any OSA characteristics (all p>0.10; **see Table S3**). *APOE4* carriers had significantly lower overall AHI, RDI, and ODI; REM AHI, as well as lower NREM AHI and ODI when compared to *APOE4* non-carriers (all p<0.05; **see Table S4**), indicating that in this cohort, *APOE4* carriers did not have greater OSA severity compared to non-carriers. There was also no significant difference in OSA characteristics among participants with and without a parental history of AD (all p>0.30; **see Table S5**). These findings indicate that groups with higher AD risk did not show evidence of greater OSA severity in this cognitively intact cohort.

### Sex, age, *APOE4 status*, parental history of AD effects on verbal memory

As previously reported, females had higher RAVLT scores than males (all p<0.05; **see Table S6**)[47]. Age and verbal memory were not significantly correlated (all p>0.10; **see Table S7**) and RAVLT scores did not significantly differ between *APOE4* carriers and non-carriers (all p>0.20; **see Table S8**). Interestingly, participants with parental history of AD performed better across the RAVLT compared to those without parental history of AD (all p<0.002; **see Table S9**).

### Associations between sleep apnea and verbal memory and total learning

**Figure 1:**
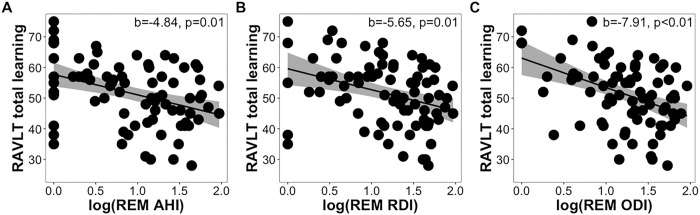
Scatter plots showing (A) AHI, (B) RDI, and (C) ODI during REM sleep as they relate to RAVLT total learning scores while controlling for age, sex, time between assessments, years of education, and *APOE4*status.

Total AHI (b=−4.47, p=0.09), RDI (b=−3.70, p=0.17, and ODI (b=−5.17, p=0.09) were not significantly associated with total learning. Similar results were observed when substituting *APOE4* status with parental history of AD. When testing OSA metrics by sleep stage, we found that REM AHI (b=−4.84, p=0.01, FDR-corrected p=0.07), REM RDI (b=−5.65, p=0.01, FDR-corrected p=0.06), and REM ODI (b=−7.91, p=0.001, FDR-corrected p=0.02) were all significantly associated with total learning, with REM ODI as the only predictor surviving FDR correction (Figure 1A–C). In the models with parental history of AD as the covariate instead of *APOE4* status, REM RDI and REM ODI were significant predictors of total learning after FDR correction (all FDR-corrected ps<0.05). However, these same features in NREM sleep were not significantly associated with total learning performance after adjusting for covariates (NREM AHI: (b=−1.32, p=0.63), NREM RDI: (b=−1.47, p=0.56), NREM ODI: (b=−2.41, p=0.40)). In addition, WASO, TST nadir and average oxyhemoglobin desaturation, and duration spent with <90% blood oxyhemoglobin saturation in TST, NREM, and REM were not significantly associated with total learning (all p>0.40). Similar insignificant results were found with models that included parental history of AD instead of *APOE4* status.

**Figure 2:**
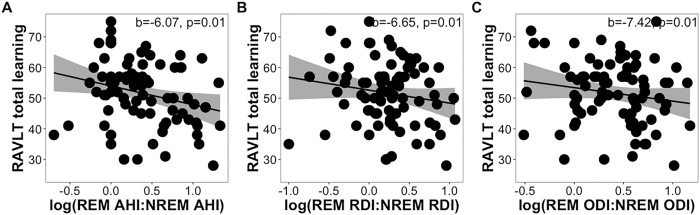
Scatter plots showing REM-NREM (A) AHI, (B) RDI, and (C) ODI ratios as they relate to RAVLT total learning scores while controlling for age, sex, time between assessments, years of education, and *APOE4*status.

Steiger’s Z test revealed that correlations between total learning and AHI (z=1.66, p=0.10), RDI (z=1.29, p=0.20), and ODI (z=1.51, p=0.13) did not differ significantly between REM and NREM sleep. Multiple regression models revealed that the ratios of REM-NREM AHI (b=−6.07, p=0.01), RDI (b=−6.65, p=0.01), and ODI (b=−7.42, p=0.01) were significantly associated with total learning (Figure 2A–C). Consistent results were found in models with parental history of AD instead of *APOE4* status as the covariate (all p<0.02). These findings indicated that greater OSA severity during REM sleep in comparison to NREM sleep was associated with diminished total learning performance.

### Associations between sleep apnea and short-delay recall

Overall AHI (b=−0.84, p=0.27), RDI (b=−0.44, p=0.56), and ODI (b=−0.80, p=0.37) were not significantly related to short-delay recall. REM AHI (b=−1.38, p=0.01, FDR-corrected p=0.21) and REM ODI (b=−1.54, p=0.03, FDR-corrected p=0.23) were associated with short-delay recall, but were no longer significant after FDR correction. In models with parental history of AD instead of *APOE4* status, REM AHI was associated with short-delay recall (p=0.04; FDR-corrected p=0.48), but not after FDR correction. Steiger’s Z test revealed a significant difference in the correlation strengths between short-delay recall and AHI during REM sleep versus during NREM sleep (z=2.81, p=0.005), demonstrating that REM AHI was more strongly associated with short-delay recall than NREM AHI. Moreover, multiple regression models indicated that REM-NREM AHI (b=−2.38, p<0.001), RDI (b=−1.77, p=0.02), and ODI (b=−2.02, p=0.03) ratios were negatively associated with short-delay recall, both in models featuring *APOE4* status and parental history of AD covariates (all p<0.03). Thus, individuals with more severe sleep apnea had worse short-delay recall, particularly if OSA was more prevalent during REM sleep as opposed to during NREM sleep.

### Associations between sleep apnea and long-delay recall

**Figure 3:**
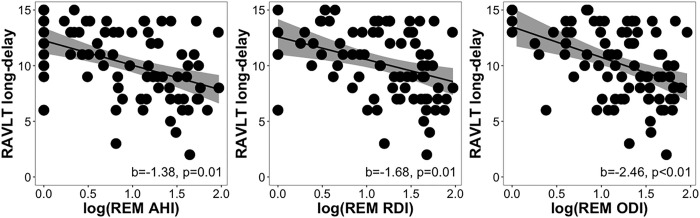
Scatter plots showing (A) AHI, (B) RDI, and (C) ODI during REM sleep as they relate to RAVLT long-delayed scores while controlling for age, sex, time between assessments, years of education, and *APOE4*status.

Total AHI, RDI, and ODI were not significantly associated with long-delay recall (p>0.07). However, REM AHI (b=−1.38, p=0.01, FDR-corrected p=0.07), REM RDI (b=−1.68, p=0.01, FDR-corrected p=0.05), and REM ODI (b=−2.46, p=0.001, FDR-corrected p=0.02) were all significantly associated with worse long-delay recall (Figure 3A–C). In models using parental history of AD instead of *APOE4* status as the covariate, REM AHI, REM RDI, and REM ODI remained significant predictors (FDR corrected p<0.05). Demonstrating specificity, these same OSA parameters during NREM sleep were not significantly predictive of long-delay recall (NREM AHI (b=0.07, p=0.93), NREM RDI (b=−0.70, p=0.93), NREM ODI (b=−0.08, p=0.93). Nadir and average oxyhemoglobin desaturation during total sleep, duration spent with <90% blood oxyhemoglobin saturation across total sleep and in NREM and REM sleep stages, and WASO were also not significant predictors (all p>0.12). Similar insignificant results were found with models with NREM OSA severity predicting long-delay recall that included parental history of AD instead of *APOE4* status.

**Figure 4:**
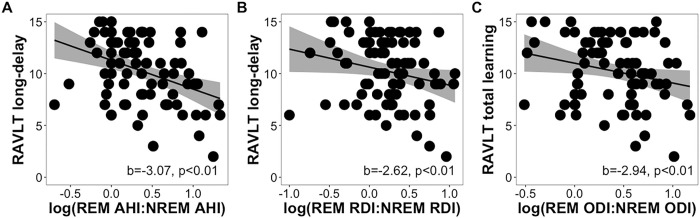
Scatter plots showing REM-NREM (A) AHI, (B) RDI, and (C) ODI ratios as they relate to RAVLT long-delayed scores while controlling for age, sex, time between assessments, years of education, and *APOE4*status.

Steiger’s Z tests revealed significant differences in the correlation strengths between long-delay recall and REM AHI (z=2.90, p=0.004) and REM ODI (z=2.14, p=0.03) versus NREM features, but not in RDI (z=1.85, p=0.07), indicating that the frequency of events and extent of oxyhemoglobin desaturations in REM sleep were more strongly associated with long-delay recall than the number of NREM respiratory disturbances. Further, multiple regression models showed that the ratios of REM-NREM AHI (b=−3.07, p<0.001), RDI (b=−2.62, p=0.001), and ODI (b=−2.94, p<0.001) were significantly negatively associated with long-delay recall (Figure 4A–C). Results from models with parental history of AD in place of *APOE4* status were similar (all p<0.003). Collectively, these findings suggest that worse verbal memory learning and recall performance were specifically associated with greater OSA severity during REM sleep and not during NREM sleep.

As a control analysis, we analyzed whether periodic limb movement of sleep index (PMLSI) was associated with RAVLT performance. We found that there was no significant association between PLSMI and verbal memory (all p<0.06).

### The moderating effects of AD risk factors on verbal memory

**Figure 5:**
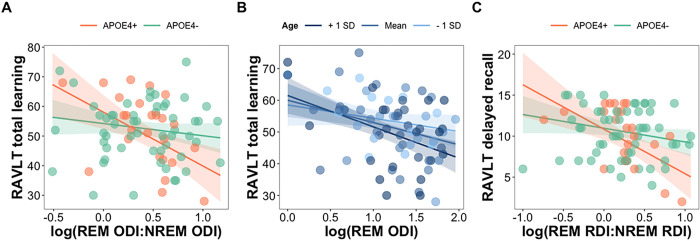
(A) The association between REM-NREM ODI ratio and RAVLT total learning scores was significantly moderated by APOE4 status. Only the *APOE* e4 allele carriers showed that more oxyhemoglobin saturations during REM sleep as opposed to NREM sleep was related to worse learning performance. (B) The association between REM ODI and RAVLT total learning scores was moderated by age. A significant moderating effect was present at the mean age and 1 SD above the mean age (in 80% of the sample). (C) The association between REM-NREM RDI ratio and RAVLT long delay recall score was moderated by *APOE4* status. A significant moderating effect was observed for *APOE4* carriers in that more respiratory events during REM sleep than in NREM sleep was associated with fewer words remembered after a 20-min delay.

Next, we characterized the moderating influence of sex, age, and genetic and familial risk of AD on relationships among OSA variables and verbal memory measures. We found that *APOE4* carriers demonstrated a significant association between REM-NREM ODI ratio and total learning (b=−18.17, p<0.01) as opposed to non-carriers (b=−4.12, p=0.17; Figure 5A). In models with parental history of AD as the covariate, age significantly moderated the association between REM ODI and total learning (b=−0.47, p<0.05). There were significant effects at the mean age and at 1SD above the mean age (all p<0.01; Figure 5B), with 80% of the sample in the significant moderating range **(Figure S1)**. Taken together, these findings indicate that the negative impact of oxyhemoglobin desaturations during REM sleep (relative to NREM sleep) on RAVLT total learning was more pronounced in those that were *APOE4* carriers and in those aged 60 or older. Further, the REM-NREM RDI ratio was significantly associated with long-delay recall in *APOE4* carriers (b=−5.42, p<0.001) but not in those without *APOE4* (b=−1.67, p=0.06; Figure 5c). This suggests that the association between higher REM-NREM RDI and long-delay recall was specific to individuals with genetic risk for AD.

Lastly, we binned participants into the 3 groups based on presence/absence of *APOE4* status and parental history of AD: 1) people with no AD risk factors, 2) people with either *APOE4* status or parental history of AD, and 3) people with both AD risk factors. We then used ANCOVA models to probe interactions between OSA characteristics and AD risk factor groups as they related to verbal memory. In individuals with both AD risk factors, we found that higher REM RDI (b=−14.08, 95%CI:[−21.53, −6.63], REM-NREM RDI ratio (b=−23.20, 95%CI:[−35.34, −11.06] and REM-REM ODI (b=−23.49, 95%CI:[−35.46, −11.51]) were significantly associated with worse total learning (**See Figure S2 and Table S8-S12** for contrast testing). Similiarly, in individuals that were both *APOE4* positive and had parental history of AD, higher REM-NREM RDI ratio was significantly associated with lower long-delay recall (b=−8.27, 95%CI:[−12.13, −4.42]; **See FigureS3 and Table S13** for contrast testing results). Overall, these findings suggest that more OSA-related events in REM sleep (relative to NREM sleep) strongly impaired word list learning and recall, especially for those that had both parental and genetic risk for AD.

## Discussion

In this study, we assessed the relationships between OSA features and verbal memory performance, and tested the moderating effects of biological sex, age, *APOE4* status, and parental history of AD on these relationships. We found that greater OSA severity during REM sleep was associated with worse verbal learning and delayed memory recall in a cohort of cognitively unimpaired middle- and older-aged adults enriched for AD risk. Additionally, more oxyhemoglobin desaturations during REM sleep versus NREM sleep were associated with worse learning performance, specifically in those that were older than 60 years old and *APOE4* carriers. Further, more respiratory events and arousals during REM sleep, as opposed to during NREM sleep, had a greater negative impact on recall performance for those those who were APOE4 carriers. The negative effects of OSA during REM sleep, specifically respiratory disturbances and oxygen hemoglobal desatursations, on verbal memory was most prominent in those that had both a parent with AD and was an *APOE4* carrier. Since AD risk factors (e.g., female sex, older age, or genetic or familial risk) were not associated with more severe OSA in this current study, these findings were not simply driven by increased OSA severity in individuals with AD risk factors. Though it is possible this could be related to lower survival from conversion to mild cognitive impairment (MCI) or AD in older adults with AD risk and more severe OSA[9,13,48,49]. That being said, our results supports the hypothesis that the memory consequences of OSA are particularly important for cognitively intact older adults with AD risk factors (older age, *APOE4* positivity, and parental history of AD), particularly when OSA events occur during REM sleep.

OSA predominantly expressed in REM sleep is a common condition and REM-sleep related physiological changes lead to increased susceptibility to airway collapse, with longer durations of apnea and hypopnea and more severe oxyhemoglobin desaturations[25,26,50,51]. This is consistent with our findings that demonstrated higher AHI, RDI, and ODI scores during REM sleep relative to NREM sleep, with REM OSA events more strongly linked with verbal memory performance than NREM OSA events. However, future investigations comparing samples enriched for more severe REM or NREM OSA are needed to determine whether it is specifically REM OSA severity that negatively impacts verbal memory performance.

REM OSA may impact verbal memory learning and recall via active disruption of memory processing or through long-term damage to brain structures and brain network function relevant for memory processing during REM sleep. While considerable attention has been given to the role of NREM sleep features in memory processing[52], there is evidence that REM sleep also supports memory. Neuroplastic processes needed for both memory consolidation and forgetting has been observed during REM sleep, in addition, hippocampal replay also occurs during this sleep stage[53,54]. Further, behaviorally, REM sleep has been linked to both emotional and spatial navigational memory[55]. Metabolic demand is also greater during REM sleep as opposed to wake and NREM sleep, including in memory-relevant regions, such as the MTL[23,24]. Therefore, OSA events in REM sleep could potentially cause memory deficits through both i) transient disruptions in cerebral gluclose metabolism in memory networks actively supporting memory processing during REM sleep and ii) long term degeneration of memory networks resultant from the presence of repeated hypoxia during high metabolic demand.

Varga and colleagues demonstrated the acute cognitive consequences of REM OSA in which they found that when withdrawing positive airway pressure (PAP) treatment specifically during REM sleep, spatial memory performance was reduced when compared to continued PAP treatment during REM sleep[22]. Although, the impact of withdrawing treatment during NREM sleep was not assessed, these findings indicated that REM OSA could cause transient MTL dysfunction by actively disrupting memory formation and consolidation even prior to neurodegenerative processes.

Hippocampal atrophy has also been reported in people with OSA, which could explain the memory impairments observed in OSA [5–9]. REM-related hypoxia and sleep fragmentation could possibly accelerate the neurodegeneration and cognitive decline via a vascular mechanism[56–60]. REM sleep is characterized by increased sympathetic activation, decreased vagal tone, and cardiovascular instability, and REM OSA has been linked to poor cardiovascular health[26,56–58]. While REM sleep has shown to have high cerebral blood flow in memory-releveant brain areas, REM OSA severity has also been associated with reduced regional cerebral blood flow in those regions [60,61]. In addition, cardiovascular risk factors have been linked to impaired memory functioning in older adults[62]. Thus, it is possible that the compounded effects of REM OSA and vascular dysfunction greatly increases oxidative stress, neuroinflammation, blood brain barrier breakdown, and/or endothelial dysfunction causing neurodegenerative-associated memory deficits in older adults[26,63].

We found that AD risk factors including older age and both parental and genetic risk for AD all exacerbated the effect of OSA severity during REM sleep on memory function. While it has been reported that the associations between OSA and cognition are weaker in older age, our findings suggested in contrast, that the relationship between oxygen desatursations in REM sleep and verbal memory were actually strongest in older individuals[9]. As our cohort consisted of individuals with undiagnosed OSA and we are unaware of the true age of OSA onset, it is quite possible that some of the older participants may have had untreated OSA longer than the younger participants. We thus cannot discount that our findings may be more related to the consequences of the duration of untreated OSA than age of OSA onset, per se.

While it is unclear whether *APOE4* status increases risk for SDB, our findings suggest that *APOE4* carriers may be more vulnerable to the impact of OSA, especially during REM sleep, on memory function. Other studies have reported similar findings in that, in *APOE4* carriers, OSA severity was associated with worse memory and executive function and had increased odds of cognitive decline[64–67]. Furthermore, disrupted sleep and *APOE4* status may synergistically exacerbate expression of hallmark AD pathologies of amyloid and tau [68,69].

The combined effects of parental history of AD and *APOE4* positivity has shown to have strong negative effects on learning and memory[70,71]. In addition, older age, family history of AD, and *APOE4* status have been linked to a smaller hippocampus and greater accumulation of amyloid and tau pathologies[72,73]. Moreover, in a subset of this cohort, we found that increasing age was related to elevated cerebreal spinal fluid (CSF) markers of tau phosphorylation and neuroinflammation, which were then associated with impared sleep-dependent memory[32]. This points to the possibility that accumulation of AD pathologies will be intensified by REM-related OSA leading to poor memory function, with the effects strongest or even just specific to those that are older and with parental and/or genetic risk for AD. Alternatively, REM OSA may contribute to cognitive impairment through cerebrovascular disease, and may be a contributor to the common comorbidity of AD and vascular dementia [74]. Prospective studies will be necessary to investigate whether REM OSA accelerates expression of AD pathologies or contributes to cognitive impairment through cerebrovascular dysfunction, or both, as well as why individuals with AD risk factors and OSA may be more cognitively impaired.

We did not find sex-specific effects in the associations between OSA and memory. In this specific cohort, males presented with more severe NREM and REM OSA, and had worse verbal memory performance than females. The lack of a sex effect could be due to a cognitively healthy sample overall that included females with a less severe OSA presentation. While females have increased risk for AD and present with greater levels of pathological tau in AD sensitive regions compared to males, it is possible that the negative effects of OSA on verbal memory performance may be more exaggerated only once women are tau and/or amyloid positive, due to the female verbal memory advantage[16,17,47,75,76]. In support of this possibility, this cohort average age was <65 years old and, based on CSF assessment, a subsample of 58 participants were almost entirely β-amyloid and tau negative [32]. As others have reported, once females progress from MCI to AD dementia, there is a steeper memory decline compared to males[18]. Future investigations are needed that combine multimodal neuroimaging, sleep apnea testing, and other memory measures, like visuospatial memory, to examine this in more detail. Regardless, it is of note that despite the cohort being largely β-amyloid and tau negative, AD risk still remained a significant moderator of OSA-memory relationships, indicating that these effects cannot be entirely explained by and may even precede β-amyloid positivity, despite findings from a recent report[77].

Some limitations of this study should be addressed. This was a cross-sectional study that found correlational relationships between OSA characteristics during REM sleep and verbal memory. Longitudinal studies will be necessary to examine how treatment of REM-related OSA would affect memory decline and progression to MCI or AD. Further, the memory testing and sleep measurements did not typically occur on the same day. While we controlled for time between measurements, this study does not directly address memory processing that occurs over a night of sleep, but rather informs upon sleep abnormalities and memory associations at the trait level of individual differences. Another limitation is that this study had exclusions of multiple medications that are commonly taken by older adults, which could potentially bias the sample and reduce the generalizability of the results. It is also important to note that our cohort was mostly White (88%) and that our findings may not be generalizable, since racial/ethnic disparities and differences exist for both OSA and AD risk factors and the relationships between these risk factors are not well studied in underrepresented populations[78,79].

These findings further support the possibility that OSA could be a modifiable risk factor for AD, and that treatment might reduce risk for cognitive decline in people vulnerable to AD[9]. This is further supported by previous study findings that showed continuous positive airway pressure (CPAP) adherence decreases the odds of AD dementia and slowed cognitive decline[80–82]. Importantly, a systematic review reported that PAP treatment adherence covers mostly the first half of the night, which could potentially leave much of REM sleep OSA untreated, since REM sleep dominates the latter half of the night[83]. It will be critical for future investigations to examine whether more aggressive OSA treatment that covers the entire sleep period would mitigate cognitive impairment and AD risk in individuals with OSA. With growth of the aging population, there is a need for interventions targeting prevention of MCI and AD, and early diagnosis and effective treatment of OSA may be one approach that could reduce risk for neurodegenerative diseases and cognitive dysfunction associated with AD.

## Conclusion

In conclusion, these findings suggest that more severe OSA during REM sleep and more REM OSA events as opposed to NREM OSA events were linked to worse verbal memory performance. This relationship was particularly true for older adults and individuals with a genetic risk for and parental history of AD. This suggests that the negative memory consequences of OSA, specifically when OSA events occur during REM sleep, are particularly impactful in individuals with multiple AD risk factors. The sleep-stage specific findings emphasize the importance of a thorough OSA screening with sleep recording capable of assessing sleep-stage specific expression of OSA, as certain individuals may have high REM AHI, while presenting with a low overall AHI. This is particularly important given that most ambulatory, non-PSG methods for assessing OSA do not include the capacity to assess REM versus NREM sleep specific OSA expression. Without sleep-stage dependent characterization of OSA, individuals that are more susceptible to memory decline, especially those with AD risk factors, may miss the opportunity to be referred for comprehensive neuropsychological evaluation and aggressive OSA treatment that may delay cognitive decline and/or AD onset.

## Figures and Tables

**Table 1.** Participant Descriptive Characteristics (n=81)

	Mean [SD]
**Demographics**	
PSG age (years)	61.68 [6.0]
RAVLT age (years)	61.38 [6.13]
Time between PSG and RAVLT	0.31 [0.50]
Female (n; %)	49; 60%
*APOE* ε4-positive genotype (n; %)	26; 32%
Parental history of AD (n; %)	56; 69%
Both *APOE* ε4-positive genotype and parental history of AD	21; 26%
Years of education	16.43 [2.41]
**Primary Race**	
White (n; %)	71; 87.8%
Black or African American (n; %)	9; 11.1%
Asian (n; %)	1; 1.2%
**Ethnicity**	
Non-Hispanic (n; %)	79; 97.5%
Hispanic (n; %)	1; 1.2%
Unknown (n; %)	1; 1.2%
	
**RAVLT**	
Total Learning	51.63 [10.48]
Short-Delayed Recall	10.77 [2.89]
Long-Delayed Recall	10.22 [3.17]

PSG = polysomnography; RAVLT = Rey Auditory Verbal Learning Test; *APOE* = apolipoprotein E; AD = Alzheimer’s Disease; *APOE4* genotyping missing from one participant;

**Table 2.** Participant Sleep Architecture Characteristics (n=81)

	Mean [SD]
**Sleep Architecture**	
Total time in bed (minutes)	451.81 [65.50]
Total sleep time (minutes)	339.30 [72.92]
Sleep onset latency (minutes)	22.98 [24.04]
Sleep efficiency (%)	75.64 [14.35]
Wake after sleep onset (minutes)	89.54 [59.70]
Stage NREM 1 (%)	9.59 [9.91]
Stage NREM 2 (%)	59.99 [10.80]
Stage NREM 3 (%)	13.26 [10.31]
Stage REM (%)	17.03 [6.32]
Apnea-Hypopnea Index (AHI)	8.26 [12.34]
REM AHI	17.19 [20.99]
NREM AHI	6.42 [12.15]
REM AHI:NREM AHI	4.02 [4.55]
Respiratory Disturbance Index (RDI)	15.96 [16.58]
REM RDI	24.19 [22.58]
NREM RDI	14.20 [16.65]
REM RDI:NREM RDI	2.53 [2.40]
Oxyhemoglobin Desaturation Index (ODI)	12.69 [15.30]
REM ODI	23.80 [21.10]
NREM ODI	10.41 [15.39]
REM ODI:NREM ODI	3.60 [2.97]
Nadir Blood Oxyhemoglobin saturation (%)	85.62 [7.98]
Mean Blood Oxyhemoglobin saturation (%)	94.78 [1.69]
Duration of <90% Blood Oxyhemoglobin saturation (minutes)	4.37 [9.24]
REM <90% Blood Oxyhemoglobin saturation (minutes)	1.83 [4.57]
NREM <90% Blood Oxyhemoglobin saturation (minutes)	2.55 [7.21]
Periodic Limb Movement Index	15.33 [19.37]

REM = rapid eye movement; NREM = non-rapid eye movement

## Data Availability

The data are available upon reasonable request and can be obtained by completing a Wisconsin Alzheimer’s Disease Research Center resource request: https://www.adrc.wisc.edu/apply-resources.

## Abbreviations

AD: Alzheimer’s disease
ADRC: Alzheimer’s Disease Research Center
AHI: Apnea-hypopnea Index
ANCOVA: Analysis of covariance
APOE4: Apolipoprotein E ε4
BMI: Body mass index
CPAP: Continuous Positive Airway Pressure
CSF: Cerebral spinal fluid
FDR: False Discovery Rate
hdEEG: High-density electroencephalography
MCI: Mild cognitive impairment
MTL: Medial temporal lobe
NREM: Non-rapid eye movement
ODI: Oxyhemoglobin Desaturation Index
OSA: Obstructive Sleep Apnea
PAP: Positive airway pressure
PLMSI: Periodic leg movements during sleep index
PSG: Polysomnography
RAVLT: Rey Auditory Verbal Learning Test
RDI: Respiratory Disturbance Index
REM: Rapid eye movement
SBD: Sleep Disordered Breathing
TST: Total sleep time
TIB: Time in bed
WASO: Wake after sleep onset
